# Supplementary material for: Positional Distribution of Fatty Acids in Processed Chinook Salmon Roe Lipids Determined by 13C Magnetic Resonance Spectroscopy (NMR)
Source: Molecules. 2023 Jan 3;28(1):454. doi: 10.3390/molecules28010454 (PMC9824307; doi:10.3390/molecules28010454)
Supplement: Supplementary file 1 [file molecules-28-00454-s001.zip › molecules-2098874-supplementary.pdf]

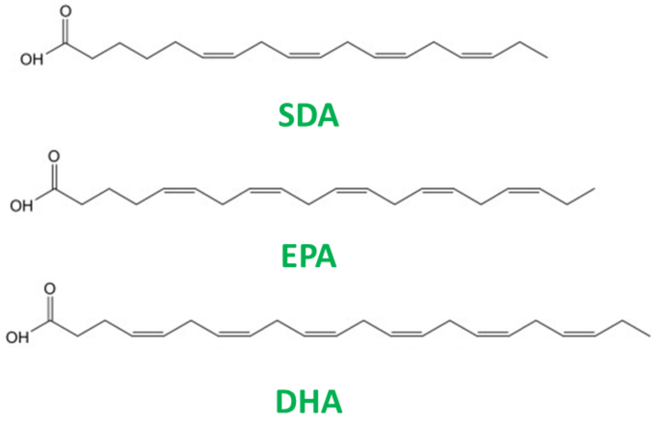

Figure S1: Structure of major n-3 fatty acids.

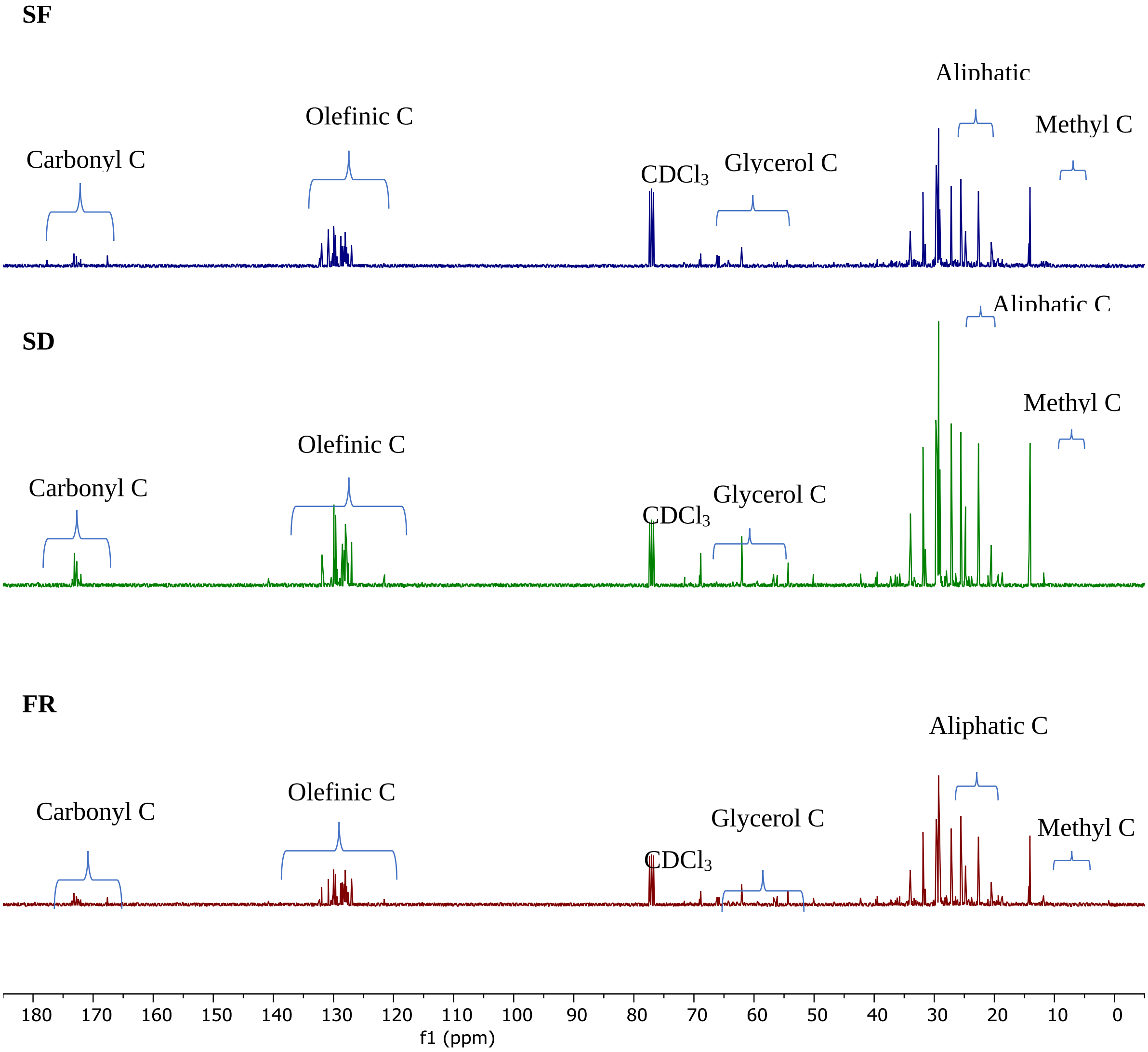

Figure S2. ^{13}C NMR full spectra of lipid extracted from fresh (FR), salted dried (SD) and fermented (SF) salmon roe. A) FR salmon roe lipid; B) SD salmon roe lipid; C) SF salmon roe lipid. Abbreviations: FR= fresh roe; SD= salted-dried; SF=salted-fermented; and C= carbon

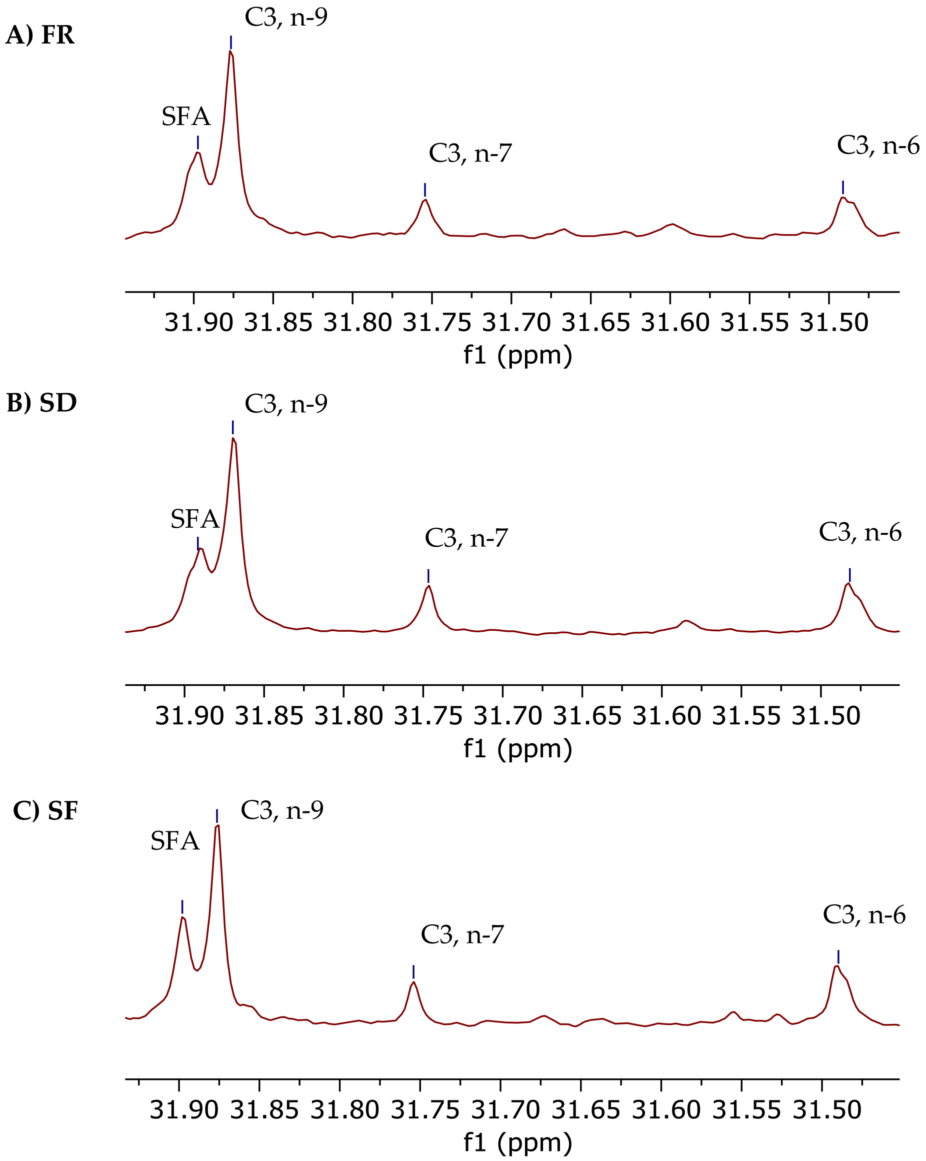

Figure S3: Aliphatic region of lipid of fresh, salted and fermented salmon roe by 13C-NMR. The region where carbon number 3 (C3) gives signal from the methyl end. A). FR= fresh salmon roe lipid extract; B). SD= salted-dried salmon roe lipid extract; C). SF=salted-fermented salmon roe lipid extract. Chemical shift (δ) regions were 32.0 – 31.0 ppm.

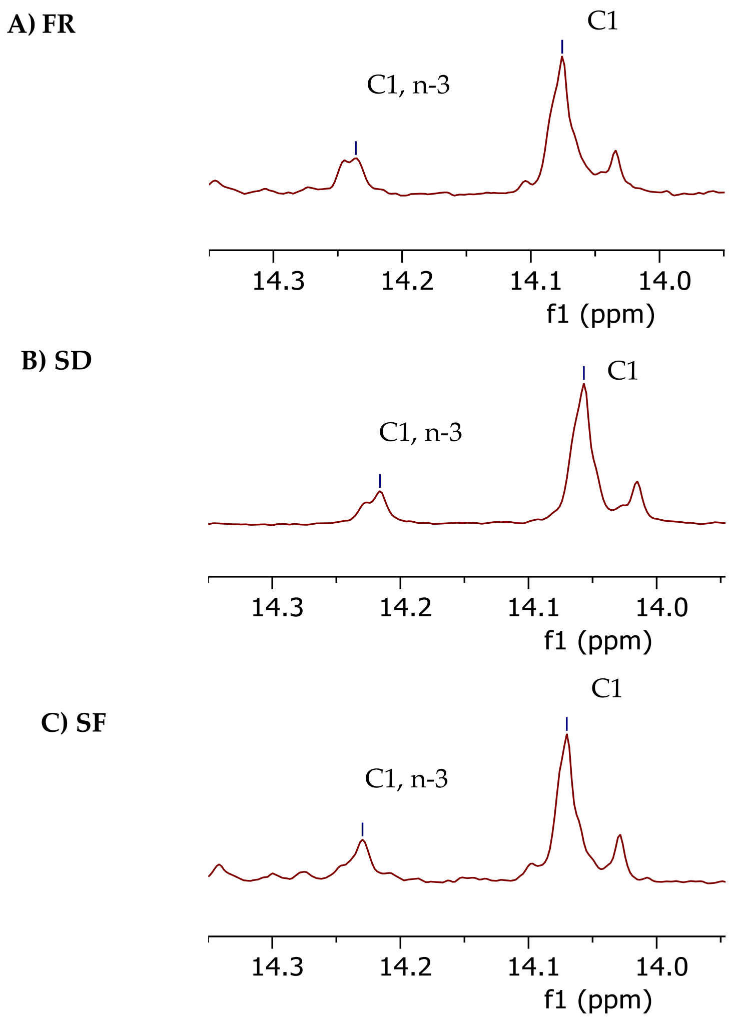

Figure S4. Expansions of 13C-NMR spectra of lipids extracted from fresh, salted and fermented salmon roe in the methyl end region, where C1 gives a signal. A). FR= fresh salmon roe lipid extract; B). SD= salted salmon roe lipid extract; C). SF= Fermented salmon roe lipid extract. δ = chemical shift (ppm).

Table S1. The regions and assignments of ^{13}C-NMR that can be noticed from the spectra of salmon roe lipids.

	Regions	Functional groups	Chemical shift
a)	carbonyl atoms region	-C=O	174.3 – 172.6 ppm
b)	double bonds/olefinic region	-C=C-	132.0 – 126.0 ppm
c)	glycerol backbone region	-COO-CH3	72.0 – 60.0 ppm
d)	aliphatic group region	-(CH2)n-	32.0 – 20.0 ppm
e)	chain ending methyl group region	$-CH_3$	~14.0 ppm
